# Reducing Mis-triage in Emergency Departments (RemEDy): Protocol for Improving Triage Accuracy Through Real-time Evaluation and Artificial Intelligence

**DOI:** 10.2196/92264

**Published:** 2026-05-27

**Authors:** Serena Sibilio, Bastiaan Van Grootven, Annette Christine Mettler, Philippe Claude Cattin, Ursula Feuz, Michael Simon, Franziska Zúñiga, Christian Hans Nickel

**Affiliations:** 1 Department of Public Health, Institute of Nursing Science Faculty of Medicine University of Basel Basel, Basel-City Switzerland; 2 Department of Public Health KU Leuven Leuven, Flanders Belgium; 3 Department of Biomedical Engineering University of Basel Allschwil, Basel-Landschaft Switzerland; 4 Emergency Department University Hospital of Basel Basel, Basel-City Switzerland; 5 Research Unit for Emergency Medicine Odense University Hospital Odense Denmark

**Keywords:** triage evaluation, emergency department, mis-triage, artificial intelligence, AI, decision support, training program

## Abstract

**Background:**

Mis-triage represents a global concern, with reported rates ranging from 15% to 33%. Understanding its causes and contributing factors is essential for ensuring patient safety. Currently, available studies have mainly focused on evaluating triage systems rather than investigating the human factors affecting triage performance. A major limitation in triage evaluation studies is the lack of standardized criteria to assess patient acuity and the absence of a clear consensus on how to measure triage accuracy. Most studies rely on retrospective data, which often fail to capture real-life clinical complexity. Therefore, the underlying causes and consequences of mis-triage remain partially understood.

**Objective:**

This study aims to improve triage by defining the optimal triage evaluation process and identifying clinician-, patient-, and system-level factors that compromise its accuracy and safety.

**Methods:**

Reducing Mis-Triage in Emergency Departments (RemEDy) will be a 4-phase, mixed methods project conducted across 7 Swiss emergency departments. The first phase will focus on developing a standardized triage evaluation instrument, combining evidence from a scoping review of triage evaluation processes, workshops with triage clinicians using design thinking methodology, and a modified Research and Development–University of California Delphi involving international experts and patient representatives. The second phase will prospectively implement this instrument in real time within a multicenter observational cohort study to evaluate triage performance; quantify mis-triage; and identify predictors at the patient level (eg, demographics), clinician level (eg, training), and system level (eg, crowding and length of stay). The third phase will focus on designing and validating an artificial intelligence–based decision support tool, applying multimodal models that integrate real-time triage data to enhance acuity prediction and minimize human error. The fourth phase will develop and evaluate a targeted training program, guided by the Capability, Opportunity, Motivation, and Behavior model, to strengthen triage accuracy and mitigate cognitive biases.

**Results:**

The project was funded by the Swiss National Science Foundation in March 2025 (grant 10004535). At submission, the scoping review is ongoing and expected to be completed in early 2026. Development and piloting of the triage evaluation instrument will take place in 2026. A multicenter cohort study is planned between October 2026 and June 2027. The intervention study is scheduled between October 2027 and December 2028. Final results are expected in 2029.

**Conclusions:**

The RemEDy project addresses key limitations of current triage research, including the lack of standardized evaluation methods. By combining expert and clinician consensus; real-time assessment; and multilevel analysis of patient-, clinician-, and emergency department–level factors, RemEDy is expected to provide a more comprehensive understanding of mis-triage and its causes. RemEDy will establish a novel framework for real-time triage evaluation and inform the development of targeted training programs with the potential to improve triage accuracy, safety, and equity.

**International Registered Report Identifier (IRRID):**

PRR1-10.2196/92264

## Introduction

The demand for emergency care varies and can change rapidly. Emergency department (ED) performance depends on the number of patients who present and on their flow through the ED to their safest and most appropriate disposition. Effective triage is critical to ensure that patients receive the right care at the right time, in the right place, and with the right resources [1,2]. However, resources have not been able to keep up with growing demand for emergency care, leading to frequent overcrowding in EDs worldwide and making accurate triage even more crucial to ensure patient safety [3-7]. Despite the use of validated triage systems such as the Emergency Severity Index, mis-triage remains a major concern in EDs worldwide, with rates reported between 14.5% and 33% [8-10]. Understanding the causes of mis-triage and its contributing factors is not only essential for ensuring safe and equitable patient management but also for maintaining the delicate balance of ED operations. Although several attempts to explore this phenomenon have been made [8,11-14], they have not provided a complete understanding of its causes and consequences, and the factors associated with mis-triage remain incompletely understood. Current studies on triage have often focused on the triage system itself, overlooking how human factors, such as the triage clinician’s cognitive biases and decision-making behaviors, contribute to mis-triage [15-18].

Additionally, a major issue in triage evaluation studies and mis-triage identification is the lack of standardized criteria for assessing patient acuity and the absence of a clear consensus on how to evaluate triage performance [19]. Most triage studies have assessed acuity either using criteria specified by expert opinion or using outcomes such as mortality or intensive care unit (ICU) admission. Although these studies provide important insights into triage evaluation, they do not accurately reflect real-world triage practices or the true causes of mis-triage. Most of these surrogate outcomes do not fully capture the heterogeneity of situations encountered in triage practice and fail to capture patients who should not wait, such as those with severe pain from non–life-threatening conditions (eg, renal colic) [20]. Criterion validity studies (eg, patient urgency as defined by experts) may include information collected throughout the entire ED visit [13]. However, they are often influenced not only by the subjectivity of the assessment, as shown by their varying interrater agreement rates, but also by their retrospective nature [21]. Furthermore, most current triage studies largely rely on retrospective data, electronic health records (EHRs), simulated scenarios, or written clinical vignettes [8,12,22-25]. These methods often fail to capture the complexity and variability of real-life situations. Written cases tend to simplify the dynamics of communication and patient assessment in the ED. Additionally, the information they provide is preselected and structured to provide what is necessary for code assignment, whereas in most cases, information is actively gathered during triage practice. This further detaches the assessment of the causes of mis-triage and the evaluation of triage performance from the realities of actual clinical practice. Therefore, a major issue in triage evaluation and mis-triage identification is the lack of standardized criteria for assessing patient acuity and the absence of a clear consensus on how to evaluate triage performance. Consequently, there is a need for a standardized triage evaluation instrument capable of delivering a comprehensive and structured assessment of triage performance.

Finally, recent advances in artificial intelligence (AI) offer promising opportunities to enhance triage accuracy by supporting clinicians during decision-making [26-32]. AI tools have shown potential in predicting key outcomes, such as hospital admission and mortality, based on data collected at triage, and may support and improve the human decision-making process [30]. However, the extent to which they support triage equity and reduce mis-triage is still underinvestigated and therefore requires further investigation in real-world settings [17,18].

To address these knowledge gaps, it is essential to investigate not only triage systems but also the performance of triage clinicians in real time in the ED setting. Therefore, a novel, prospective approach to triage evaluation is necessary to reflect the complexity of everyday triage decision-making and to inform targeted strategies for reducing mis-triage. Therefore, the Reducing Mis-Triage in Emergency Departments (RemEDy) project aims to improve triage by (1) defining the optimal triage evaluation process by creating a novel triage evaluation instrument; (2) conducting prospective data collection in real-life ED triage settings to understand the actual causes of mis-triage to identify contributing factors and reduce them; (3) determining how AI can contribute to improving triage decisions, ensuring that these decisions are as equitable as possible, and how models can be improved for use in everyday ED practice; and (4) developing and evaluating a triage improvement training program to reduce mis-triage of patients in ED.

## Methods

### Ethical Considerations

At this stage of the project, ethics approval is not applicable. Ethics approval and informed consent will be obtained for all subsequent project phases involving human participants, as required by local and national regulations.

### Study Design

**Figure 1:**
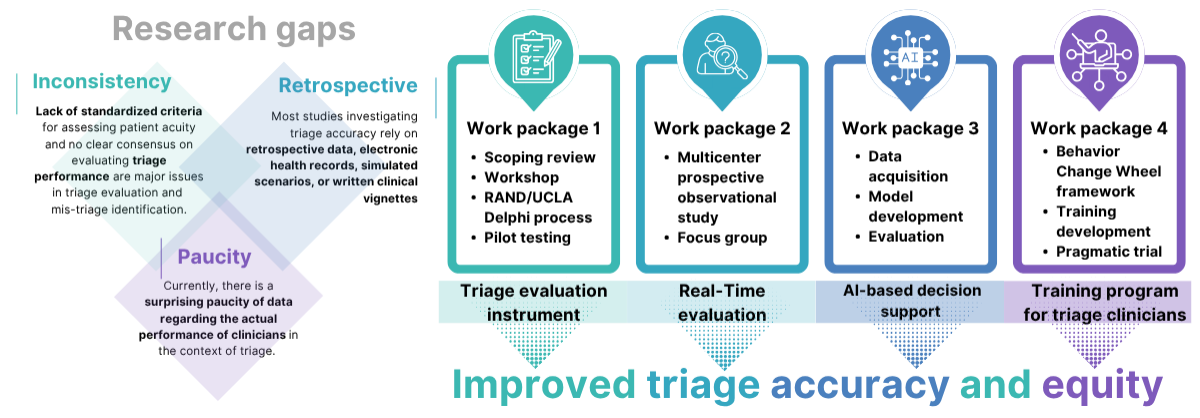
Overview of identified research gaps and the Reducing Mis-Triage in Emergency Departments project, divided into 4 phases.

The RemEDy project is a multiphase mixed methods project encompassing 4 phases, each designed to address a specific subaim within the broader objective of improving triage performance in EDs (Figure 1). The study will involve 7 EDs in Switzerland and will use both quantitative and qualitative methodologies.

### Development of a Standardized Triage Evaluation Instrument

The objective of the first phase of the project is to gain a comprehensive understanding of mis-triage and its consequences. We will identify and consolidate mis-triage definitions, review current methods for evaluating triage processes, and examine the available outcomes used to assess triage performance. To achieve this, a scoping review will first be conducted following the Joanna Briggs Institute methodology [33]. Study selection and data collection will be conducted independently by at least 2 researchers. Study flowcharts, data coding manuals, relevant software (eg, Covidence [Veritas Health Innovation] and Zotero [Corporation for Digital Scholarship]), and pilot and calibration exercises will be used to ensure study quality [34,35]. The PRISMA-ScR (Preferred Reporting Items for Systematic Reviews and Meta-Analyses Extension for Scoping Reviews) guidelines will be used to report the study [36].

Findings of this review will initially be evaluated in a workshop with a minimum of 8 selected triage experts. The workshop will be based on design thinking methodology [37], which allows the development of user-centric solutions through iterative processes, collaboration, and prototyping. In this first workshop, the overarching aim is to understand the user’s needs (in this case, triage clinicians) and the fundamental elements of a triage evaluation process. This will help identify the needs and challenges associated with triage evaluation by gathering information directly from relevant stakeholders, such as triage nurses, physicians, and decision-makers in EDs. The initial mapping of the triage evaluation tool will be conducted using storytelling activities, brainstorming, and cocreation techniques (eg, mind mapping). An initial set of potential prototype elements of the triage evaluation instrument will be developed in this workshop.

Subsequently, a panel of experts consisting of 40 national and international triage experts will be invited to participate in 2 online rounds of a modified Delphi process to assess the appropriateness and feasibility of the triage evaluation instrument’s potential prototype elements. The Research and Development–University of California (RAND-UCLA) Appropriateness Method will be selected for its well-established and robust technique for assessing the appropriateness of medical practices and interventions [38]. Additionally, patient representatives from the European Patients’ Academy on Therapeutic Innovation in Switzerland will be recruited to give feedback during all stages of the Delphi process. In the first round, experts will be asked to rate the triage evaluation instrument’s potential prototype elements on a 9-point Likert scale for 2 dimensions: appropriateness (ie, the extent to which the element supports accurate and effective triage evaluation) and feasibility (ie, how practical it is to consider or implement the element during real-life triage evaluation). After the first round, we will analyze ratings to identify areas of consensus and disagreement. Items that require discussion based on the ratings will be summarized, and a steering committee, composed of members of the research team and 10 to 15 selected national and international experts, including patient representatives, will address these issues for further refinement. In the second round, the potential prototype elements will be redistributed to the expert panel for re-evaluation. Participants will receive anonymized aggregated data from the previous round, including the group’s median ratings and comments. This structured feedback encourages participants to reconsider their positions and fosters convergence toward consensus. Consensus will be based on the Research and Development–University of California Appropriateness Method.

Once the key elements for the triage evaluation instrument have been identified, the revised prototype will be operationalized using the design thinking methodology in a second workshop [37]. This workshop will include developed personas for triage evaluation, applying the revised prototype. The “fit” of the revised prototype across different triage evaluation scenarios will be explored, and potential gaps in the evaluation will be identified and resolved by discussing triage evaluation scenarios.

The full prototype will then be piloted in the University Hospital of Basel in approximately 50 patient triage encounters, involving at least 5 health care professionals working in triage. Triage experts will be asked to give feedback on usefulness and feasibility, as well as to suggest improvements to the instrument through structured interviews. The full prototype instrument will be revised where necessary based on the results of the pilot testing and expert feedback.

A key methodological consideration of the RemEDy project is that subsequent phases depend on the development of the triage evaluation instrument in phase 1. Although this introduces an element of uncertainty regarding the final outcome, this sequential design is intentional and reflects the current lack of standardized methods for evaluating triage performance. To address this, the instrument will be developed using a multistep process, including a scoping review, expert consensus, and pilot testing of the triage evaluation tool. The instrument will be implemented in subsequent phases only after successful piloting.

### Identify and Analyze Mis-Triage in Real Time

The aim of the second phase of the project is to identify and analyze mis-triage in a real-time ED setting by evaluating routine triage procedures and comparing them with expert triage assessments using the triage evaluation instrument. A 7-center prospective cohort study will be conducted, in which routine triage will be evaluated by triage experts using the developed instrument. Expert triage decisions will serve as the reference standard for evaluating triage accuracy. Experts will be selected based on predefined criteria, including a minimum of 5 years of clinical experience in ED triage, formal triage training, and active involvement in clinical practice. In addition, all triage experts will undergo standardized training on the triage evaluation instrument and participate in calibration exercises using clinical scenarios to ensure consistent application of the evaluation criteria prior to data collection.

Triage experts will observe routine triage and use the evaluation instrument to document the correct triage level (considered the gold standard) as well as the reasons for mis-triage. Routine triage level assignments will be compared with the gold standard (triage experts) to identify correct triage and mis-triage, which will be further specified as undertriage and overtriage.

### Sample Size Considerations

An initial sample size calculation was conducted to inform resource planning. Using the *pmsampsize* package in R (version 4.5.2; R Foundation for Statistical Computing), we estimated that a minimum of 2152 participants would be required to develop a model with up to 20 predictor degrees of freedom, assuming a prevalence of undertriage of 20% and a target C statistic of 0.7. During the project, this sample size will be further refined to account for clustering at the triage clinician level when more information becomes available. On the basis of administrative data and stakeholder consultations, we expect an average recruitment rate of 10 eligible patients per working day, with 2 shifts per week over approximately 50 working days. We anticipate minor variation in recruitment rates across sites and expect to complete the recruitment within a 6-month time frame. An interim review of recruitment and sample distribution across centers will be considered to confirm feasibility and determine whether adjustments to the recruitment period or sample size are needed. The final sample size will be determined after completion of the predictor selection process. As the required sample size depends on the number of predictors included in the model, the current estimate will be refined once the final set of variables has been established.

### Data Collection

Two key analyses will be conducted. First, predictors of overtriage and undertriage will be explored. Predictors will include patient characteristics (such as patient demographics, presenting symptoms, vital signs, mode of arrival, clinical history, and frailty score) collected from EHRs; triage clinicians’ characteristics (such as years of experience in triage and in the ED, triage training history, degree, years of experience in other departments, and demographic information) obtained through online survey prior to the start of the study; and ED metrics, such as occupancy rate (eg, total number of patients physically present in the ED divided by the number of available treatment spaces at a given time), and ED length of stay of the patients collected from administrative data.

For this phase of the project, the study protocol will be submitted to the relevant ethics committee for approval, and appropriate informed consent will be obtained from participating patients in accordance with local and national regulations.

### Data Analysis

Data analysis will help identify patient groups and characteristics at risk of mis-triage, as well as clinician traits and ED performance metrics (such as crowding) associated with a higher likelihood of mis-triage. At 30 days, we will conduct an evaluation of selected outcomes for all enrolled patients, as done previously [39-42]. A multivariable multinomial logistic regression model will be fitted with the outcomes of correct triage, undertriage, and overtriage, and predictors at the patient level will be tested. Predictors at the level of triage clinicians and the ED will be explored by extending the previous model to a generalized linear mixed-effects model.

The consequences of mis-triage will also be explored. Patient outcomes related to mis-triage will be documented by the research staff in Research Electronic Data Capture (REDCap) based on information in the EHR. Outcomes of interest include 30-day mortality, hospitalization, ICU admission, number of resources needed, as well as the set of outcomes identified through the scoping review and selected during the development of the triage evaluation instrument [43]. There will be 2 end points, one during the ED stay and the other at 30 days. To determine whether triage errors are linked to a higher risk of adverse outcomes, the sample will be divided into three groups: (1) correct triage, (2) undertriage, and (3) overtriage. Cases of undertriage and overtriage will be reviewed by an expert panel within 30 days to determine the clinical impact of mis-triage. An incidence analysis will be performed stratified by triage group. Group differences will be tested with a chi-square test, and risk ratios for undertriage and overtriage will be calculated for each outcome.

Finally, an explanatory sequential mixed methods design will be embedded in this study. Focus groups with triage clinicians working in the 7 centers will be organized to clarify identified associations in the statistical models and determine behavioral and contextual determinants of improvement [44]. An interview guide will be drafted to explore questions related to triage experiences and to elicit interpretations of observed associations in the predictors and consequences of mis-triage.

At the time of submission of this protocol, the cohort study has not yet started, and no data have been collected. Preparation of the detailed study protocol and submission to the relevant ethics committees will be initiated during the first semester of 2026. Patient recruitment and data collection for the prospective multicenter cohort study are planned to take place between October 2026 and June 2027. Analysis of cohort data and evaluation of outcomes are expected to commence after completion of data collection.

### Development of an AI Algorithm to Support Triage Decision-Making

The objective of the third phase of the project is to improve triage quality and equity by developing AI support tools to reduce mistakes when human errors in triage occur and when the currently used triage algorithms lead to mis-triage by design.

A *base model* will be developed using existing emergency patient data before the start of the multicenter study and will be applied during the study. Well-established classifier models, such as gradient-boosted decision tree models, including Extreme Gradient Boosting, will be used to predict the triage scores (triage levels 1-5, as defined by clinicians) and odds of mis-triage by the model. International guidelines for trustworthy and deployable AI in health care [45] will be followed to avoid bias and ensure fairness and explainability. An *advanced model* will be developed and validated with data acquired in the observational study. Multimodal models similar to Ecole Polytechnique Federale de Lausanne’s Modular Clinical Decision Support Networks will be explored and combined with a *continuous learning model* approach to make them more robust against domain shifts caused by, for example, changing demographics or other external factors. Continuously learning models offer the opportunity to adapt to changes in data distribution over time. How to adapt these approaches to tabular and multimodal data is still being researched. However, recent developments, such as MedFuncta and TabINR, are promising starting points on how to combine multimodal and tabular data [46,47].

Model performance will be measured by comparing (1) the model’s prediction with the clinician’s triage classification and (2) the model’s performance with the gold standard triage decisions provided by the experts. Outcome measures include accuracy, area under the curve, *F*_1_-score (a metric in machine learning and statistics to evaluate the accuracy of classification models), sensitivity, and specificity. The *F*_1_-score is the harmonic mean of precision and recall, reflecting a balance between the model’s accuracy in positive predictions and its completeness in identifying positives. It is particularly important because it is independent of any data imbalances (ie, certain classes being underrepresented in the dataset). The availability of expert triage decisions allows for detecting whether the model outperforms “routine” triage decisions.

This phase will be conducted in parallel with the multicenter prospective cohort study described in phase 2.

### Development and Testing of a Triage Improvement Training Program

Finally, the project aims to develop and evaluate a triage improvement training program to reduce mis-triage in the ED. Triage decision-making will be conceptualized as a complex behavior, and the Behavior Change Wheel methodology, underpinned by the capability, opportunity, motivation, and behavior model, will guide the development [44]. The training will be developed through workshops with triage clinicians, and a survey will evaluate appropriateness and acceptability before implementation.

A pragmatic trial with a 2-group parallel design will be used to evaluate the effectiveness of the training program in reducing mis-triage at the University Hospital of Basel. An initial sample size calculation was conducted. We hypothesize a reduction in mis-triage from 30% to 20%, which requires a sample size of 294 patients per group to achieve 80% power with a significance level (α) set at .05. However, because patients are clustered within triage clinicians and cluster sizes may be unequal, the final sample size will be updated using estimates from the phase 2 cohort study. Specifically, we will use cohort data to (1) estimate the overall prevalence of mis-triage, (2) estimate the intraclass correlation at the clinician level (and any higher-level clustering, if present), and (3) summarize the distribution of patients per clinician. These estimates will be used to finalize the sample size calculation. Recruitment targets will then be adjusted accordingly.

A generalized estimating equations model will be used to evaluate the effectiveness of the training program in reducing mis-triage.

Subsequently, an embedded feasibility study will be conducted. A random subgroup of triage clinicians will crossover (5 clinicians per group) to a group having access to the AI triage algorithm. Clinicians will be asked to use the triage algorithm for 3 shifts (10×3=30 shifts in total) to investigate the feasibility (defined as “the extent to which a new treatment, or an innovation, can be successfully used or carried out within a given agency or setting”), acceptability (defined as “the perception among implementation stakeholders that a given treatment, service, practice, or innovation is agreeable, palatable, or satisfactory”) [48], and suggestions for improvement. Triage clinicians’ perspectives on the algorithm’s usability and its future implementation in clinical settings will be explored through structured interviews.

At the time of submission of this protocol, the training intervention study has not yet started, and no data have been collected. Preparation of the detailed study protocol and submission to the relevant ethics committees will be undertaken prior to initiation of the intervention phase. Implementation of the triage improvement training program and associated data collection are planned to take place between October 2027 and December 2028. Analysis of trial data and evaluation of training effectiveness and feasibility will be conducted following completion of data collection.

## Results

The RemEDy project was funded by the Swiss National Science Foundation in March 2025 (grant 10004535). At the time of submission of this protocol, the scoping review is ongoing, and no results have been generated. Study selection and data extraction are currently in progress. Data collection for the scoping review is expected to be completed in early 2026. Development of the triage evaluation instrument, including design thinking workshops, the Delphi consensus process, and pilot testing, is planned throughout 2026, with completion expected by September 2026. These activities will be conducted after completion of the scoping review. The first work package is expected to be completed by September 2026, after which results from this phase of the project will be available.

The multicenter prospective cohort study, involving 7 Swiss EDs and a target sample size of approximately 3147 patients, is scheduled between October 2026 and June 2027. Development and validation of AI models will be conducted in parallel with the cohort study using both retrospective and prospective data. The intervention study, including a pragmatic trial evaluating a triage training program, is planned between October 2027 and December 2028.

## Discussion

### Anticipated Findings

The expected output of the RemEDy project will be an evidence-based triage evaluation instrument that sets a new benchmark for assessing triage and provides insights into the consequences of mis-triage. The derived instrument will allow for real-time triage evaluations in different ED settings. Hence, the results of this project will address critical shortcomings of current triage practices, particularly those influenced by human factors [49]. We will gain insight into knowledge gaps among triage clinicians, limited competencies, and issues related to clinical judgment and behavioral aspects, as well as cognitive biases or implicit prejudices, such as those related to age, sex, race, or comorbidities that may impact triage outcomes. We will be able to describe patient-related factors, such as disease presentation, comorbidities, and physiological changes, and environmental factors, such as occupancy rates and patient length of stay, and examine their relationship with mis-triage [50,51].

Through real-time data collection across multiple EDs, it will be possible to identify patient predictors associated with mis-triage and measure the real clinical impact of incorrect triage decisions. Current knowledge about triage practices relies on retrospective evaluations or outcomes that fail to adequately capture the essential goal of triage, which is to accurately identify patients who should not wait to be seen. A literature review by Kuriyama et al [13] on existing measures for the validation of triage systems reported that 51 out of 57 studies used construct rather than criterion validity, and the most used outcomes were hospitalization, ICU admission, ED mortality, or mortality at 7 or ≥30 days. Another study [11] has assessed triage performance using outcomes such as mortality and hospitalization rates or by using certain critical illnesses (eg, sepsis or pulmonary embolism) as benchmark conditions [52]. Approaches based on criterion validity, including expert assessments of patient urgency, can leverage data accumulated throughout the entire ED stay. However, these methods are dependent on clinician interpretation and are applied retrospectively, which may introduce variability and limit their ability to provide consistent and unbiased evaluations [21]. Hence, none of the *construct-* or *criterion-based* triage studies available to date have adequately identified the root causes of mis-triage and its contributing factors. Additionally, a recent study conducted by Sax et al [10] assessed the frequency of mis-triage using the Emergency Severity Index in a cohort of >5 million patients across 21 hospitals. This retrospective cohort study created operational definitions for each Emergency Severity Index level using EHR data from ED visits to classify encounters as undertriaged, overtriaged, or correctly triaged. The retrospective nature of the study does not allow a full understanding of what led to the misclassification of patients and the real causes of mis-triage [20]. For example, a patient presenting with nonspecific symptoms may be undertriaged due to a misleading clinical presentation, highlighting how diagnostic uncertainty can contribute to mis-triage. In a recent literature review conducted by Tam et al [24], written case scenarios and retrospective review were commonly used to examine triage accuracy. These methods do not allow a detailed understanding of the decision-making process underlying triage, as they focus on outcomes rather than the cognitive and contextual factors influencing clinical judgments. Furthermore, the information contained in these texts is preselected and structured to provide what is necessary for code assignment, whereas in most cases, information is actively gathered during triage practice. This further separates the assessment of the causes of mis-triage and the evaluation of triage performance from the realities of routine clinical practice.

A strength of RemEDy is its first-time use of real-time prospective, multicentric observational data to evaluate triage performance, establishing a new standard for comparing future triage evaluation studies. Using a multidimensional approach, RemEDy will study triage, including the examination of clinicians’ behaviors and biases, to identify the key predictors of poor triage performance and its clinical impact; once identified, these predictors will be integrated by AI into an unbiased computer-based clinical decision aid designed to ensure consistent, equitable triage performance. After independent external validation in several Swiss EDs, training programs for the decision aid to assist its deployment into clinical practice will be developed and evaluated. In addition, the RemEDy project will include experts from diverse disciplines, such as emergency medicine, nursing science, and biomedical engineering, as well as patient representatives. This multidisciplinary and multiprofessional approach will ensure that the research is relevant and directly applicable to clinical practice, contributing to improving triage practices within EDs.

One expected limitation relates to the generalizability of findings outside the Swiss ED context. Although the study primarily includes 7 diverse centers providing different levels of care, local clinical routines, staffing structures, local policies, and triage practices might influence the assessment of triage practices. However, the use of standardized data collection tools and calibration methods will enhance measurement validity and replicability in other settings. Furthermore, the involvement of international experts in the Delphi process, along with the observation that many ED populations share comparable characteristics across countries, will support the interpretation and adaptation of our findings to other health care systems.

Another limitation may arise from expert recruitment and participation in the consensus and piloting phases. To address this, international professional societies and networks will be involved early in the planning process. Another potential limitation concerns the risk of systematic measurement errors that could bias study results. To mitigate this, standardized operating procedures will be applied across all study tasks, supported by structured training sessions and calibration exercises for involved personnel, alongside regular data quality checks and monitoring visits. Finally, the development and validation of AI tools in this project will require ongoing refinement to ensure fairness, transparency, and performance across subpopulations. These models will function solely as decision support tools and will not influence real-time clinical decision-making until full validation is achieved.

In summary, the RemEDy project aims to establish novel benchmarks for triage evaluation and to inform the development of future triage practices and policies. The findings of this project will be disseminated through peer-reviewed publications, presentations at international conferences, and professional magazines in different EDs.

### Conclusions

This project was designed to address key gaps in the evaluation of triage performance by enabling standardized and real-time assessment of triage processes. Through its prospective and multidimensional approach, the project will generate evidence on the determinants and consequences of mis-triage in routine clinical practice. The findings are expected to contribute to a better understanding of the factors influencing triage accuracy and to inform the development of future strategies aimed at improving safety and equity in emergency care.

## Abbreviations

AI: artificial intelligence
ED: emergency department
EHR: electronic health record
ICU: intensive care unit
PRISMA-ScR: Preferred Reporting Items for Systematic Reviews and Meta-Analyses Extension for Scoping Reviews
REDCap: Research Electronic Data Capture
RemEDy: Reducing Mis-Triage in Emergency Departments
